# Neuromuscular disorders: genes, genetic counseling and therapeutic trials

**DOI:** 10.1590/1678-4685-GMB-2016-0019

**Published:** 2016

**Authors:** Mayana Zatz, Maria Rita Passos-Bueno, Mariz Vainzof

**Affiliations:** 1 Human Genome and Research Center (HUG-CELL), Instituto de Biociências, Universidade de São Paulo (USP), São Paulo, SP, Brazil.

**Keywords:** genetic diseases, genetic counseling, neuromuscular disorders, stem cells, therapies

## Abstract

Neuromuscular disorders (NMD) are a heterogeneous group of genetic conditions, with autosomal dominant, recessive, or X-linked inheritance. They are characterized by progressive muscle degeneration and weakness. Here, we are presenting our major contributions to the field during the past 30 years. We have mapped and identified several novel genes responsible for NMD. Genotype-phenotype correlations studies enhanced our comprehension on the effect of gene mutations on related proteins and their impact on clinical findings. The search for modifier factors allowed the identification of a novel "protective"; variant which may have important implication on therapeutic developments. Molecular diagnosis was introduced in the 1980s and new technologies have been incorporated since then. Next generation sequencing greatly improved our capacity to identify disease-causing mutations with important benefits for research and prevention through genetic counseling of patients' families. Stem cells researches, from and for patients, have been used as tools to study human genetic diseases mechanisms and for therapies development. The clinical effect of preclinical trials in mice and canine models for muscular dystrophies are under investigation. Finally, the integration of our researches and genetic services with our post-graduation program resulted in a significant output of new geneticists, spreading out this expertise to our large country.

## Introduction

Neuromuscular disorders (NMD) include a wide group of genetic conditions that affect about 1 in 1000 individuals worldwide. They are characterized by progressive muscle degeneration and weakness due to genetic mutations which primarily or secondarily impair skeletal muscle function. Most of these mutations display autosomal recessive, autosomal dominant or X-linked inheritance. The onset can occur in childhood and have a severe progression or later in life with a slower course. A complete list of diseases/genes/phenotypes is available at http://www.musclegenetable.fr/.

Our group, at the Institute of Biosciences, at the University of São Paulo, was pioneer in establishing a center for diagnosis, genetic counseling and research in neuromuscular disorders in the late 1970s. In the 1980s, we founded the Brazilian Muscular Dystrophy Association, which has recently joined with a larger entity for handicapped patients, the AACD. In 2000, the Human Genome Research Center was founded, and in 2005 stem cells research was introduced, to the now called Human Genome and Stem Cells Research Center (HUG-CELL). To date, we have attended about 26,000 patients with NMD and at-risk relatives at the HUG-CELL. Here, we will summarize our main contributions to the field.

## Mapping and identification of new genes

Although the human genome project was declared completed in 2003, it has been recently estimated that only 50% of the 7.315 Mendelian phenotypes, usually represented by rare disorders, have been associated to given genes (Chong *et al.*, 2015). This scenario is even more complex and challenging for disorders with multifactorial inheritance, in which multiple variants, genes, environmental effects and epigenetic phenomena may be involved and are usually highly heterogeneous. Therefore, the identification of putative causative variants of clinical phenotypes is still an important approach in human genetics, providing a direct link between a particular phenotype and a given gene.

We have mapped 8 loci responsible for neuromuscular disorders and identified for the first time five of their mutational gene mechanisms. We have also collaborated with the identification of another five genes.

Large families with many affected members, as well as isolates with high degree of consanguinity were very important to identify new disease genes (Figueiredo *et al.*, 2015).

### Duchenne Muscular Dystrophy

In 1981, we described a case of a girl affected by Duchenne muscular dystrophy (DMD), carrying an X-autosomal translocation with a breakpoint at Xp21 (Zatz *et al.*, 1981). This case, together with similar cases reported in the literature, was key to confirm the mapping of the Duchenne locus at Xp21, leading to the identification of the DMD gene in 1988 by the group of Louis M. Kunkel (Koenig *et al.*, 1998; Monaco *et al.*, 1988).

### Limb-girdle muscular dystrophies

Subsequently we focused on limb-girdle muscular dystrophies (LGMD), a group of disorders that affect primarily the pelvic and scapular limb-girdles. LGMD can be transmitted through autosomal recessive (AR) and, less frequently, autosomal dominant (AD) inheritance. Currently there are 19 AR (LGMD2A-2T) and eight AD genes (LGMD1A-1H) already identified. Affected patients can have a mild disease course remaining ambulant until late in life or have a severe phenotype, clinically very similar to X-linked DMD. Among the severe forms, LGMD2C, LGMD2D, LGMD2E and LGMD2F are sarcoglycanopathies, caused by mutations in the SGCG, SGCA, SGCB and SGCD genes, coding for γ-SG, α-SG, β-SG, and δ-SG, components of the sarcoglycan (SG) complex. These transmembrane glycoproteins, together with sarcospan, dystrophin, dystroglycans, syntrophins and α-dystrobrevin, constitute the Dystrophin-glycoprotein complex. This complex acts as a linker between the cytoskeleton of the muscle cell and the extracellular matrix, providing mechanical support to the plasma membrane during myofiber contraction (Yoshida and Ozawa, 1990). Among patients diagnosed with AR LGMD (through DNA and/or muscle protein analysis) from 120 families in Brazil, the LGMD2A is the most frequent form of sarcoglycanophaty (Zatz *et al.*, 2003).

The first LGMD gene identified by our group is responsible for LGMD2F, caused by mutations in δ-SG gene coding for one of the four sarcoglycan proteins. This gene causes a severe childhood form of muscular dystrophy (Nigro *et al.*, 1996; Passos-Bueno *et al.*, 1996). Another gene mapped and identified by our group is responsible for AR-LGMD2G, caused by a mutation in the TCAP gene, coding for the protein telethonin (Moreira *et al.*, 1997, 2000). Although identified in compound heterozygous patients belonging to a family of Italian origin, LGMD2G has been shown afterwards to be very rare, and few cases have been reported outside Brazil. LGMD2G is characterized by a variable phenotype, with onset in childhood or adulthood (Vainzof *et al.*, 2002).

The protein telethonin is a 19 kDa component of the sarcomere Z-disk in striated and cardiac muscles (Valle *et al.*, 1997). Telethonin is the first example of a sarcomeric protein, of which deficiency was associated with a form of muscular dystrophy, without disruption of the sarcomere structure (Vainzof *et al.*, 2002).

More recently we have identified the gene for the AD LGMD1G muscular dystrophy (Vieira *et al.*, 2014), which had been mapped by our group 10 years earlier (Starling *et al.*, 2004). Mutations in the RNA-processing protein HNRPDL, a heterogeneous ribonucleoprotein family member, which participates in mRNA biogenesis and metabolism, were identified in two large, unrelated families: one from Brazil and the other from Uruguay. The identification of the LGMD1G gene revealed a novel association between a muscular disorder and an RNA-related gene and reinforces the importance of RNA binding/processing proteins in muscle development and muscle disease.

We also collaborated in the identification of LGMD2A (Beckmann *et al.*, 1991; Richard *et al.*, 1995; Spencer *et al.*, 1997), LGMD2B (Passos-Bueno *et al.*, 1995; Bushby *et al.*, 1996; Bashir *et al.*, 1998;), LGMD2C (McNally *et al.*, 1996b), LGMD2D and LGMD2E genes (Bonnemann *et al.*, 1996), responsible for several forms of AR-LGMDs (Zatz *et al.*, 2003).

### Spastic paraplegia (SPG)

We have mapped and/or identified several genes responsible for pure spastic paraplegia, namely SPG8 (Rocco *et al.*, 2000; Valdmanis *et al.*, 2007), SPG4 (Starling *et al.*, 2002b; Mitne-Neto *et al.*, 2007a), or X-linked SPG (Starling *et al.*, 2002a). Our group also described a new complicated autosomal recessive form of spastic paraplegia named SPOAN (spastic paraplegia, optic atrophy, neuropathy) (Macedo-Souza *et al.*, 2005, 2009). SPOAN was identified in a geographically isolated region in the backlands of Northeastern Brazil, known for a high incidence of consanguineous marriages. The mutation, recently identified through whole genome sequencing, is a homozygous deletion in the noncoding region of the kinesin light chain-2 (*KLC2*) gene, a novel mechanism, which reinforces the importance of noncoded regions in human pathology (Melo *et al.*, 2015).

### Motor neuron diseases

Motor neuron diseases are a group of neurodegenerative disorders with involvement of upper and/or lower motor neurons, such as amyotrophic lateral sclerosis (ALS), spinal muscular atrophy, progressive bulbar palsy, and primary lateral sclerosis. A new locus for a recessive X-linked juvenile form of distal muscular atrophy has also been mapped in our lab (Takata *et al.*, 2004).

However, a more important contribution from our group was the mapping and subsequent identification of the amyotrophic lateral sclerosis gene 8 or ALS8 in a very large Brazilian genealogy (Nishimura *et al.*, 2004a,b). This gene codes for the vesicle-associated membrane protein/synaptobrevin-associated membrane protein B (*VAPB*) gene. Members of the vesicle-associated proteins are intracellular membrane proteins that can associate with microtubules and that have been shown to have a function in membrane transport (Mitne-Neto *et al.*, 2007b). We have also shown that the *VAPB* mutation, which is now identified in hundreds of Brazilian patients, is due to a founder effect (Nishimura *et al.*, 2005). *VAPB* mutations have been subsequently identified in patients with different origin – from Japan and Europe (Funke *et al.*, 2010). This finding has attracted a lot of attention from researchers in the field because *VAPB* seems to be involved in other forms of ALS (Coatti *et al.*, 2015; Teuling *et al.*, 2007).

## Genotype-phenotype correlations

Neuromuscular disorders are characterized by marked phenotypic and genotypic heterogeneity, with a similar clinical course caused by mutations in different genes while several different phenotypes can be associated with mutations in one particular gene (Zatz *et al.*, 2000). Our group has contributed with this characterization for several forms of muscular dystrophies.

After the identification of the dystrophin gene as the protein involved in DMD, a correlation between dystrophin presence/quantity and the severity of the phenotype in dystrophinopathies was strongly suggested. Genotype, phenotype and protein analysis enabled us to pinpoint the important functional domains of the dystrophin protein (Vainzof *et al.*, 1993; Passos-Bueno *et al.*, 1994), and question the previously suggested correlation (Vainzof *et al.*, 1990, 1991b, c, 1995b).

For most forms of autosomal recessive LGMDs, missense mutations, on average, have been associated with a milder phenotype as compared to null mutations. (Richard *et al.*, 1999; de Paula *et al.*, 2002; Starling *et al.*, 2003). We also observed that mutations in the LGMD2I genes, first reported in severe congenital forms, could be also found in adult forms with slow progression or even asymptomatic cases (de Paula *et al.*, 2003), associated with specific protein alterations in the muscle (Yamamoto *et al.*, 2008). Muscle protein characterizations have contributed to elucidate the organization of the dystrophin-glycoprotein complex (Vainzof *et al.*, 1991a, 1996, 1999), for genotype-phenotype correlation studies and had an important role in NMD diagnosis. After the introduction of next generation sequencing (NGS), molecular diagnosis is achieved directly with DNA analysis and therefore muscle biopsies have been obtained only for research purposes.

## The search for modifier (protective) variants/factors

Genotype-phenotype correlation studies revealed that, for several disorders such as LGMD, facioscapulohumeral muscular dystrophy, the same disease mutation usually associated with a severe phenotype could be also found in individuals only mildly affected or even asymptomatic (Bonnemann *et al.*, 1996; de Paula *et al.*, 2002, 2003; McNally *et al.*, 1996a; Moreira *et al.*, 2003; Starling *et al.*, 2003; Tonini *et al.*, 2004; Ricci *et al.*, 2014; Scionti *et al.*, 2012). Although rare, this has also been observed for DMD (Zatz *et al.*, 2014; Castro-Gago, 2015; Zatz, 2015). Utrophin, an autosomal ubiquitously expressed protein with structural homology to dystrophin, has been suggested as a possible modulator of DMD severity and thus as a therapeutic target for treating DMD. However, we observed that utrophin expression does not differ between severely and mildly affected DMD patients (Vainzof *et al.*, 1995a, Vainzof 2016).

Therefore, the search for protective variants or mechanisms continues to be a great challenge. Interestingly, the identification of two golden retriever muscular dystrophy (GRMD) dogs with a very mild phenotype and a normal lifespan (Zucconi *et al.*, 2010; Zatz *et al.*, 2014) have been the subject of much investigation. We have recently shown that up-regulation of *Jagged1*, which is a known regulator of the Notch pathway, is responsible for the milder course in these two dogs (Vieira *et al.*, 2015). In addition, in the mdx mouse model for DMD, we observed a milder course associated with less fibrosis and more regeneration when the mutation was transferred to a different background (unpublished observations). Identifying what protects some exceptional dogs, mice or individuals from the deleterious effect of a disease-causing mutation is a great challenge which could open new venues for treatment (Cohn and Dubowitz, 2016).

## Genetic testing

Research in human and medical genetics allied to genetic counseling services in Brazil initiated in the late 1960s. During those years, the model implemented by Oswaldo Frota-Pessoa in our Department consisted of research associated with services to patients and families, in a mutually beneficial scenario: patients contributed to new findings, while those new findings helped patients. This model has been maintained by the geneticists at the Human Genome and Stem Cells Research Center. Genetic testing and genetic counseling in our community, has been one of the main activities of HUG-CELL with emphasis to the group of disorders associated with research projects, including: neuromuscular, neurodegenerative, craniofacial, deafness, intellectual disability, and Autism Spectrum disorders.

### From Southern blot to next generation sequencing

Molecular diagnosis for neuromuscular disorders was introduced in our center in the late 1980s based on Southern blot and PCR (polymerase chain reaction) analysis. The introduction of these methods have largely contributed for the development of research as well as for the prevention of new cases, based on diagnosis of NMD families, carrier detection in DMD/DMB families and genetic counseling. The detection of deletions in the dystrophin gene (which account for 60–70% of the mutations responsible for Duchenne dystrophy) were first performed by Southern blot, followed by multiplex analysis of the most common deleted exons by PCR. In the 1990's, the use of microsatellite markers throughout the genome led to the mapping of novel NMD genes. In addition, microsatellite markers within and flanking the dystrophin gene, allowed us to improve DMD/DMB carriers detection tests by segregation analysis, particularly in familial cases in which no deletion had been detected in the dystrophin gene. In the mid 1990's, genetic testing for diagnosis of Myotonic muscular dystrophy and Facioscapulohumeral disorder were introduced, both based on Southern blot methodology. In 2000, with the inauguration of the Human Genome Center, a non-profit DNA diagnostic lab was installed. The acquisition of a semi-automated Sanger sequencing equipment opened the possibility to again expand and improve genetic testing (by allowing the larger scale use of Sanger sequencing methodology) and include other NMD, particularly the limb-girdle muscular dystrophies. This expansion also enabled us to reach a much wider public. In addition to benefiting patients/families seen at our center it allowed the performance of updated molecular diagnosis in DNA samples from patients throughout Brazil. Our laboratory was also one of the first core sequencing facilities at University of Sao Paulo, a service that has been offered since then and which has been continuously updated. A recent new improvement in 2013-2014 was the acquisition of next generation sequencing (NGS) equipment (MiSeq and HiSeq, Illumina) and standardization of its methodology. NGS, based on a panel of 80 genes, increased significantly the efficacy of NMD diagnosis, with molecular alterations being identified in ~73% of the cases. In addition to decreasing the cost of NMD testing, the use of a panel of genes avoids the ethical issues associated to the identification of incidental findings through exome sequencing. NGS has also imposed a great challenge in bioinformatics training and expertise development on how to deal, store and analyze large data sets.

Whole exome sequencing (WES) which was standardized in our center in 2014 has brought important contributions to basic research, and to identification of new disease loci and pathogenic mutations. WES also enables a better characterization of the Brazilian population genetic variability, which is crucial for interpreting sequence analysis and for obtaining accurate diagnosis (Zatz *et al.*, 2012) (http://laboratorio.genoma.ib.usp.br; http://genoma.ib.usp.br).

## Genetic counseling

Genetic counseling (GC) is of upmost importance for the prevention of genetic diseases, especially for the untreatable ones. The process of GC includes diagnosis confirmation, identification of at-risk members, prenatal and pre-implantation diagnosis, as well as family orientation on management of affected patients.

Several ethical issues regarding the use of genetic tests have been the subject of much debate since the pre-molecular era, particularly regarding asymptomatic at-risk relatives in late-onset disorders. In accordance with an international consensus, we do not test asymptomatic children at-risk for late-onset disorders for which there is no treatment, such as cerebellar ataxias or myotonic dystrophy, as this decision should be taken solely by the subjects themselves when they reach adulthood. On the other hand, the identification of asymptomatic carriers for recessive autosomal or X-linked diseases can be crucial for the reproductive decisions of the parents.

Before offering genetic testing, several issues are discussed with patients or family members, such as which individuals should be tested, implications of test results, and how to deal with unexpected findings, such as false paternity.

More recently, the introduction of exome sequencing has opened new ethical debates particularly related to incidental findings. What should be disclosed? What is the geneticist's responsibility? Do patients and family members understand all the possibilities when signing informed consents about their willingness to be informed or not? As there is still no Brazilian regulation on this matter, we have adopted the criteria of: ACMG (American College of Medical Genetics and Genomics) Board of Directors (2013). We provide information to those who are interested about the at-risk variants associated with disorders, to which preventive measures and/or treatment are available. Our experience has shown that there is no rigid rule and each case has to be discussed by the genetic counseling team before deciding how to better approach the patients' family.

Most services in Brazil offer prenatal diagnosis (PND) in spite of abortion not being allowed for genetic disorders reasons (the only exception for a legal abortion is the confirmation of anencephaly). In our service we decided to perform PND in the 1990s for at-risk women, who would otherwise interrupt their pregnancy, fearing that their fetus could be affected by a genetic disorder (for example mothers or sisters of Duchenne muscular dystrophy patients or parents at-risk for congenital muscular dystrophy 1A, (Yamamoto *et al.*, 2004, Vainzof *et al.*, 2005). In the majority of cases referred for PND, the test results are negative for the mutation present in the family, and as a result, more women are encouraged to continue their pregnancies and unaffected babies are saved. Moreover, since many of our patients come from a poor social background, orientation on management of the genetic disease has been an important part of our service. This approach was the subject of a paper celebrating 125 years of Science magazine in 2005 (Zatz, 2005). More recently, psychoanalytical support and follow-up for patients and at-risk relatives has also been offered in our center, which has been very important particularly for patients with degenerative disorders. Finally, the integration of community services with our post-graduation program results in a significant contribution in the training of a number of geneticists, enabling them to start new Centers in other Brazilian cities and spread out this expertise to our large country.

## Stem cells: from patients and for patients

### Stem cells from patients: IPSc cells

The groundbreaking discovery of induced pluripotent stem cells (iPSCs) by Dr. Yamanaka's group in 2006 (Takahashi and Yamanaka, 2006), demonstrating the possibility to reprogram differentiated cells to an embryonic-like stem cell, opened a new venue for research. While applications of iPSCs in cell therapy are envisioned but still in a premature stage of development, the use of iPSCs as tools to study human genetic diseases mechanisms boomed in the last few years. With this in mind, we have established an IPS cells bank from patients with different neuromuscular disorders. One interesting result was obtained with patients affected by amyotrophic lateral sclerosis type 8 which had been identified by our group. We have successfully reprogrammed fibroblasts from ALS8 patients and generated motor neurons. Our results suggest that optimal levels of VAPB may play a central role in the pathogenesis of ALS8, which is in agreement with the observed reduction of VAPB in sporadic ALS and *SOD1* murine model (Mitne-Neto *et al.*, 2011).

The most recent gene editing CRISPR-cas9 technology (Doudna and Charpentier, 2014) applied to different cells derived from IPSCs will certainly bring important contributions to functional studies, enhancing our comprehension on pathological mechanisms underlying neuromuscular disorders and providing new opportunities for treatment.

### Stem cells for patients: cell therapy

#### Preclinical trials in neuromuscular and neurodegenerative disorders

The possibility to treat progressive muscular dystrophies, particularly DMD, with stem cell therapy has been of great interest and the focus of many investigations. Before starting therapeutic trials, several questions need to be addressed: What is the effect of stem-cell therapy for muscular dystrophies in animal models? What is the best source of adult stem cells? Is immunosuppression necessary? Are the experiments reproducible with different cell lines, or from different donors? Should injections be local or systemic? Most importantly, what is the safety level of non autologous stem cell transplantation? This last issue is very important for genetic conditions such as neuromuscular disorders where autologous stem cell transplantation is unlikely to be beneficial. In order to address these questions we have performed a series of pre-clinical experiments with human-derived stem cells transplanted into different murine models and GRMD dogs.

Comparison between different sources of stem cells revealed that cord tissue is a much richer source of stem cells than umbilical cord blood, and that mesenchymal stem cells from blood and tissue have a different expression profile (Secco *et al.*, 2008a,b, 2009). The relevance of this observation, which resulted in a highly cited paper, was based on the fact that umbilical cord blood banks (both public and private) had been storing blood and discarding tissue. Next, we also identified fallopian tubes as an important source of stem cells, with potential to enhance bone regeneration (Jazedje *et al.*, 2009, 2012).

Several reports on stem cell transplantation have been published by other groups in murine as well as canine models of muscular dystrophy using immunosuppressed animals (Di Rocco *et al.*, 2006; Sampaolesi *et al.*, 2006; Rouger *et al.*, 2011; Nitahara-Kasahara *et al.*, 2012). This approach may hinder the interpretation of clinical effects of stem cell injections since immunosuppressant drugs have a beneficial effect on muscular dystrophy (Davies and Grounds, 2006). Therefore, we performed our xenotransplantation experiments without any immunosuppression therapy. We first analyzed the effect of human adipose-derived stem cells (hASCs), which were injected in *SJL* mice (the murine model for dysferlinopathy). Human cells were well tolerated and treated mice performed significantly better than untreated controls in functional tests (Vieira *et al.*, 2008). We next compared the effect of human umbilical cord stem cells injected in *SJL* mice using the same protocol. Differently from the experiment with hASCs, injected mice did not show any functional improvement but untreated controls showed a decline in functional tests (Vieira *et al.*, 2010; 2012).

The next question we wanted to answer was whether observed discrepancies were due to donors' different genetic background or different stem cell sources. Therefore, we compared the effect of pericytes (which are precursors of mesenchymal stem cells, Caplan and Sorrell, 2015) derived from different tissues (adipose tissue, endometrium, abdominal muscle and fallopian tube) from a female donor undergoing hysterectomy. Pericytes were injected intraperitoneally in double-knockout mdx/utrophin mice. We observed a beneficial effect only in pericyte-injected animals, which lived significantly longer (~ 25%) (Valadares *et al.*, 2014).

#### The danger of stem cell contamination using Parkinson's disease as a model

Preliminary trials to treat highly prevalent neurodegenerative diseases, such as Parkinson's disease (PD), with mesenchymal stem cells have generated controversial results. In a rat model of PD induced by the MPTP neurotoxin, we first observed a significant bilateral preservation of dopaminergic neurons in the substantia nigra and prevention of motor deficits typically observed in PD, following intracerebral administration of human umbilical cord-derived mesenchymal stem cells (UC-MSC) early after MPTP injury. However, surprisingly, administration of fibroblasts -mesenchymal cells without stem cell properties, as a xenotransplantation control was highly detrimental, causing significant neurodegeneration and motor dysfunction independently of MPTP administration. Our pre-clinical study suggests that fibroblasts may be common cell contaminants affecting the clinical outcome in stem cell therapy protocols, which might also explain the discrepant clinical results (Pereira *et al.*, 2011). These observations should be widely disseminated, since many private clinics claim to be injecting stem cells to treat many different diseases while, in reality, it is unknown how well characterized these injected cells are.

#### Safety of nonautologous stem cell transplantation: GRMD dogs

To investigate the safety of nonautologous stem cell transplantation, we injected hASCs in the best available animal model for DMD: the golden retriever muscular dystrophy (GRMD) model. Affected animals carry a frameshift point mutation resulting in the absence of the muscle protein dystrophin (Sharp *et al.*, 1992). These dogs have a severe disease course and most do not survive beyond two years, despite some may have a variable phenotype. Although no human dystrophin was found in muscles from recipient dogs, a functional improvement was observed shortly after a series of injections followed by an apparent stabilization afterwards, without noticeable side effects. A growing body of evidence indicates that although mesenchymal stem cells are partially defined by their ability to differentiate into various tissues *in vitro,* it is their trophic, paracrine and immunomodulatory functions that may have the greatest therapeutic impact *in vivo*, decreasing inflammation and fibrosis (Murphy *et al.*, 2013; Caplan and Sorrell, 2015). This would explain the beneficial effect observed in the injected GRMD dogs. In short, we showed that repeated injections of hASCs, from different donors, are well tolerated in immunocompetent GRMD dogs. We also observed functional benefits in three dogs followed from four to six years post-transplantation, without tumor formation. This study has the longest follow-up of human cells transplanted animals ever reported. These observations, which should be replicated in larger samples, might have important applications for future therapy in patients with different forms of muscular dystrophies (Zatz *et al.*, 2015).
